# Brief culturally adapted cognitive behaviour therapy for obsessive compulsive disorder: A pilot study

**DOI:** 10.12669/pjms.314.7385

**Published:** 2015

**Authors:** Muhammad Aslam, Muhammad Irfan, Farooq Naeem

**Affiliations:** 1 Muhammad Aslam, MSc (Psychology). Psychologist, Fountain House, Lahore, Pakistan; 2 Dr. Muhammad Irfan, MBBS, MCPS, FCPS, MS. Assistant Professor and Head, Department of Psychiatry and Behavioural Sciences, Peshawar Medical College, Peshawar, Riphah International University, Islamabad - Pakistan; 3 Dr. Farooq Naeem, MBBS, MSc (Research Methodology), MRCPsych, PhD. Associate Professor of Psychiatry, Queens University, Kingston, Ontario, Canada

**Keywords:** Culturally adapted Cognitive Behavior Therapy (CaCBT), Obsessive Compulsive Disorder (OCD), Depression

## Abstract

**Objective:**

To demonstrate the effectiveness of Brief Culturally adapted Cognitive Behavior Therapy (CaCBT) in the treatment of Obsessive Compulsive Disorder (OCD).

**Methods:**

This pre and post design study was conducted on out-patients with OCD at Centre for Cognitive Behaviour Therapy, Fountain House, Lahore, from April 2011 to April 2012. A semi structured questionnaire was developed to document demographic details of all the patients. All the participants were assessed at baseline (Pre Therapy session) with Yale Brown Obsessive Compulsive Disorder Scale (Y-BOCS), Hospital Anxiety & Depression Scale (Depression Subscale & Anxiety Subscale) and Brief Disability Questionnaire (BDQ). They were re-assessed on the same scales at the end of therapy in a follow up assessment session. Feedback from patients and their family member, who were trained as co-therapist, was obtained at the end of the therapy for assessing the satisfaction with the therapy. All the patients received six sessions of brief culturally adapted cognitive behavior therapy (CaCBT). Statistical analyses were carried out using SPSS v.22.

**Results:**

The mean age of the sample (n=21) was 31.14±11.9 years. There were significant differences post CBT between the scores of Y-BOCS (p=0.000), HADS – Depression subscale (p=0.001), HADS – Anxiety subscale (p=0.000) and BDQ (p=0.000).

**Conclusion:**

This study provides preliminary evidence for effectiveness of culturally adapted CBT for OCD.

## INTRODUCTION

Obsessive Compulsive Disorder (OCD), comprises of obsessions and compulsions. “Obsessions are unwanted, unacceptable intrusive and repetitive thoughts, images, or impulses that are associated with subjective resistance, are difficult to control, and generally produce distress”.^1^ “Compulsions are repetitive, stereotyped behaviors that are usually performed in response to an obsession in order to prevent or reduce anxiety or distress”.^2^ The individual feels temporary relief by performing certain rituals or habitual ways of responding to ideas although the responses might not be logically concerned with the fears of the person’s thoughts.^3^

OCD which was considered treatment-resistant till mid-1960s is now treated with Psychotropic medications and Cognitive Behaviour Therapy (CBT).^4^,^5^ Studies with CBT suggest that in addition to being as effective as medication for the treatment of OCD^6^, it has also proven helpful in treating patients that have inadequate response to psychotropic medications. CBT including exposure and response prevention is considered an effective treatment because it focuses on the ‘here and now’ of the problem; helps in exploring and understanding alternative ways of thinking; and challenging beliefs through behavioral exercises, as opposed to other talking therapies which tend to focus on ‘past problems’.^3^,^7^ Also, CBT helps participants identify and re-evaluate beliefs about the potential consequences of engaging or not engaging in compulsive behavior, and to work toward eliminating this behaviour.^5^

Modern psychotherapies support western values as they were developed in the West. However, evidence suggests that cultural factors have a huge impact on delivery of these psychotherapies.^8^ In this context, it is believed that CBT might need modification for use in the Non Western cultures as it involves exploration and modification of automatic thoughts and core beliefs.^9^ The limited research suggests effectiveness of therapy based on CBT principles.^10^,^11^ It has been proven that it is important to culturally adapt CBT for a better effect and culturally adapted CBT (CaCBT) has been successfully used in various psychiatric conditions with promising results as cultural factors do play an important role.^12^,^13^ Lessons learned from these studies were used to culturally modify CBT for OCD in Pakistan.

CBT for OCD usually consists of 10-20 sessions and is not feasible in low and middle income country settings due to lack of resources.^14^ Therefore, brief interventions are considered acceptable and a pilot study to evaluate the effectiveness of brief CBT has shown to be effective in primary care in Pakistan.^12^

Since there is scarcity of available data of the effectiveness of CBT in OCD in low and middle income countries, this study was aimed to demonstrate the effectiveness of brief CaCBT in the treatment of OCD through a pilot study.

## METHODS

This pre and post design study was conducted on outdoor patients of OCD at Centre for Cognitive Behavior Therapy, Fountain House Lahore from April 2011 to April 2012. The ethical clearance for this study was granted by the Ethical review board of Pakistan Association of Cognitive Therapists. Twenty one patients, who fulfilled the diagnostic criteria of OCD on the basis of DSM IV-TR and were residents of Lahore city, were enrolled in this study after being provided with information about the study and written informed consent.

A semi structured questionnaire was developed to document demographic details of all the patients. All the patients were assessed for outcome measures at baseline (Pre Therapy session) with Yale Brown Obsessive Compulsive Disorder Scale (Y-BOCS), Hospital Anxiety & Depression Scale (Depression Subscale & Anxiety Subscale) and Brief Disability Questionnaire (BDQ). Depression and Anxiety are commonly associated with OCD, so Hospital Anxiety & Depression Scale (HADS) was used and since disability (functional and otherwise) is also frequently reported with OCD, BDQ was also used. The patients were re assessed on the same scales at the end of therapy in a follow up assessment session.

Y-BOCS is a 10-item scale, designed to rate the severity and type of the illness. It includes assessments of time occupied; interference with ordinary social activities; degree of distress; resistance and control; and measure symptoms, on a scale from 0 to 4.^15^

HADS is a 14 item, self-assessment scale which has been translated in Urdu and has been used widely in Pakistan.^12^ The two subscales; anxiety and depression, have been found to be independent measures. The maximum score is 21 for both depression and anxiety and is divided into normal (0-7); mild (8-10); moderate (11-15); and severe (16-21).^16^

BDQ is an 8 item questionnaire, developed by the WHO to measure disability due to physical and psychological problems. The scale has already been used in a number of studies in Pakistan.^12^,^17^

Feedback from patients and their family member, who were trained as co-therapist to help the patient at home and minimize relapse in future; was obtained at the end of therapy for assessing the satisfaction with the therapy.

Statistical analyses were carried out using SPSS v.22. Frequency and percentages were calculated for all the variables. The mean and standard deviation were calculated for the scores of all the scales used in the study. All continuous variables (for example, the pre and post scores on all the scales used) were compared using t-tests and p value obtained keeping <0.005 as the cut off point for significance.

### Culturally adapted CBT Intervention for OCD

The brief version of CBT used for this study consisted of 6 sessions, plus one additional session for the family. CBT was developed and culturally adapted using a series of qualitative studies in Pakistan. Like the standard CBT, culturally adapted CBT was delivered using a manual which emphasized on assessment and engagement, awareness of cultural factors and adjustments in therapy techniques.^18^ Intervention was culturally adapted using a series of qualitative studies in Pakistan. Information gathered from these preparatory qualitative studies, as well as our own field observations and experience of therapy and clinical practice, were collated to develop an adaptation framework that guided the CBT adaptation process.^19^ The adaptation framework was used to adapt the CBT manual. This adapted manual emphasizes on three major areas to be considered while delivering culturally sensitive therapy, i.e., assessment and engagement, awareness of cultural factors and adjustments in therapy techniques.

Few salient cultural adaptations that we incorporated in the CBT manual were:

- During the therapy sessions a member of the family accompanied the client and helped the client with homework when required
- One additional session for the whole family at the start of the therapy.
- Therapists initially focused on physical symptoms. We included physical symptoms in the fourth column in our first thought diary to highlight the importance of the physical symptoms and their association with thoughts and mood.
- Urdu equivalents of CBT jargons were used in the therapy.
- Culturally appropriate homework assignments were selected and participants were encouraged to attend even if they were unable to complete their homework.
- Folk stories and examples relevant to the religious beliefs of the local population were used to clarify issues.

Inspite of these adaptations we faced numerous barriers that are unique to this culture:

- Although the national language is Urdu that is widely spoken in Punjab Province, some clients from rural areas do not understand Urdu. Two participants from rural Punjab in this study were unable to understand Urdu. This caused difficulty in engaging with these clients. This highlights the need for further modification and adaptation of therapy for different linguistic groups in Pakistan
- Even though families were involved as part of our therapy, three female patients were stopped from seeing a male therapist
- Most patients had visited a “self-acclaimed” spiritual/faith healer, prior to visiting a mental health facility. This prolongs the duration of untreated illness. This also leads to feelings of shame and guilt, especially when the obsessions are of religious nature. In this study eight patients out of twenty one had some religious obsessions and all of them had seen a spiritual/faith healer prior to seeing a psychiatrist. A major part of the therapy therefore involved educating these patients about the biological basis of illness as well as offering them religious examples to reduce guilt.

All the patients received six sessions of brief culturally adapted cognitive behavior therapy (CaCBT). These were weekly sessions of 45 to 60 minutes, for 6 weeks. Every patient in the first session was psycho-educated and trained in breathing exercise and with exposure and response prevention. They were also asked to schedule and rate their activities routine on the form provided by the therapist, to keep them involved and to act as homework assignment. During each session, patient was encouraged to discuss events and thoughts. In addition to exposure and response prevention and breathing exercises, thought distraction; problem solving skills; and activity scheduling; taught during the course of CaCBT, helped patients to understand and analyze their thoughts, understand cognitive errors, challenge automatic negative thoughts and change these thoughts with positive alternative thoughts.

These sessions were administered in addition to psychotropic medications in nineteen patients while two patients received CaCBT only.^20^,^21^ It should be noted that telephonic contact was also used to remind home assignments and follow up visit. Patients attending less than three sessions were considered not eligible for inclusion in the analysis.

## RESULTS

Out of 21 enrolled patients, 12(57.1%) were males. The mean age of the sample was 31.14±11.9 years and most (n=9, 42.9%) were in the age group 20-29 years. Most of the patients (n=12, 57.1%) were living in joint family system. Except for one, all the patients were matriculate or above. The mean monthly income of the sample was 39785.71± 61388.2 Pakistani rupee (PKR) with all earning 50000 PKR and below except one who was earning 300000 PKR per month. The mean duration of illness was 7.67 ± 5.9 years. Only four of these (3 once and 1 twice) got admitted in a psychiatric facility in the last 12 months. Out of 21, 14 (66.7%) were diagnoses with OCD alone and 7 (33.3%) were diagnosed with OCD along with a co morbid diagnosis of Depression. The detailed demographics of the sample are given in Table-I.

**Table-I:** Demographic details of the sample (n=21).

Variable		No.	Percentage
Gender	Male	12	57.1
	Female	9	42.9
Age	< 20	2	9.5
	20-29	9	42.9
	30-39	6	28.6
	40 +	4	19.0
Family System	Joint	12	57.1
	Nuclear	9	42.9
Educational Status	Primary	1	4.8
	Matriculation	3	14.3
	Higher Secondary	8	38.0
	Graduate	6	28.6
	Masters	3	14.3
Occupation	Student	4	19.0
	Housewives	5	23.8
	Employed	9	42.9
	Unemployed	3	14.3
Monthly income in PKR	Upto 15000	6	28.6
	15001-30000	7	33.4
	30001 +	8	38.0

The results revealed that there are significant differences between the scores of Yale Brown Obsessive Compulsive Scale (p=0.000), Hospital Anxiety and Depression Scale – Depression subscale (p=0.001), Hospital Anxiety and Depression Scale – Anxiety subscale (p=0.000) and Brief Disability Questionnaire ((p=0.000)), pre and post CBT (Table-II).

**Table-II:** Pre and Post Scores (n=21).

Scale	Pre CBT	Post CBT	p–value
Y-BOCS	30.3± 6.4	9.3± 6.7	0.000
HADS- Depression Subscale	10.6± 3.7	6.4± 4.1	0.001
HADS- Anxiety Subscale	13.0± 2.8	6.3± 3.5	0.000
BDQ	11.5± 6.4	4.5± 4.9	0.000

The satisfaction with the therapy was assessed by asking four questions. Every question could have been answered in four possible options i.e., definitely not/ very dissatisfied; not really/ dissatisfied; to some extent/ satisfied; and yes, definitely/ generally. The questions and their responses are described in Table-III.

**Table-III:** Satisfaction with the therapy (n=21).

Questions	Definitely not/Very dissatisfied	Not really/Dissatisfied	To some extent/Satisfied	Yes, Definitely/Generally
Did you get the kind of support and treatment that you wanted?	-	-	2 (9.5%)	19 (90.5%)
What do you feel about the treatment in context of relieving your symptoms?	1 (4.8%)	1 (4.8%)	9 (42.9%)	10 (47.5%)
Did this treatment solve your overall problems?	1 (4.8%)	1 (4.8%)	10 (47.5%)	9 (42.9%)
Would you suggest this treatment to a friend?	-	-	3 (14.3%)	18 (85.7%)

## DISCUSSION

CBT has an established role in the treatment of OCD and Exposure and Response prevention is used as the most effective tool. In our study focusing on the same, majority were males, which is similar to a study in India^22^ but in contrast to other studies, where there is an equal ratio of both genders or females are affected at a slightly higher rate.^23^,^24^ This can very well be attributed to small sample size of our study. The mean age of the participants was 31.14±11.9 years in our study with most of the participants in the age group 20-29 years which is similar to the results shown in another Pakistani study where the mean age was 25.87± 7.39 and in majority of the subjects, the onset of OCD was before 30 years.^25^ Similar results have also been shown in various international studies.^22^,^26^ Majority of our patients were living in joint family System which is in contrast to another study in India where almost 97% of the participants were living in nuclear family system.^22^ In our study almost 95% were matriculate or above which is similar to another study from the region where almost 94% were having high school qualification or above.^22^ The average duration of illness in our study was almost seven and a half years which was more than the duration reported in other studies.^25^,^26^ In our study, one third reported having co morbid depression, the presence of which is supported in other Pakistani studies, one of which reported more than half had associated anxiety and depression.^27^

The results of our study showed significant improvement in scores of anxiety, depression and disability associated with OCD as well as in the scores of Yale Brown Obsessive Compulsive Scale, which is also shown in various studies conducted.^28^,^29^

This study also highlights the importance of culturally adapting CBT for clients from non western cultures. The content of the obsessional thoughts can be influenced by religious and cultural beliefs. Most of our patients had attended “self-acclaimed” faith healers, prior to visiting a mental health facility, who commonly misinterpret these thoughts based on their own beliefs. A major part of the therapy therefore is to educate the patient and their families about the bio-psycho-social nature of the illness. Therefore, involvement of the family is an essential part of the therapy. In spite of these modifications there were many barriers faced in therapy. Therefore there is a need for further work in order to overcome these barriers.

People undergoing CBT have reported satisfaction with the therapy. In our study, majority of patients showed satisfaction with therapy, which has already been reported in the literature.^30^

### Limitation of the study

This was a pre and post assessment study. Therefore, it provides preliminary evidence, but we cannot conclude without a randomized controlled trial that this treatment is effective.

## CONCLUSION

The low score on the post testing after receiving six sessions each on a weekly basis provides preliminary evidence for effectiveness of culturally adapted CBT in patients of OCD. The study also identifies some barriers to delivery of this treatment.
